# Correlation Between Fundus Autofluorescence Pattern and Retinal Function on Microperimetry in Choroideremia

**DOI:** 10.1167/tvst.12.9.24

**Published:** 2023-09-29

**Authors:** Federica E. Poli, Imran H. Yusuf, Jasleen K. Jolly, Laura J. Taylor, Daniel Adeyoju, Amandeep S. Josan, Johannes Birtel, Peter Charbel Issa, Jasmina Cehajic-Kapetanovic, Lyndon Da Cruz, Robert E. MacLaren

**Affiliations:** 1 Nuffield Laboratory of Ophthalmology, University of Oxford, UK; 2 Oxford Eye Hospital, Oxford University Hospitals NHS Foundation Trust, Oxford, UK; 3 Vision and Eye Research Institute, School of Medicine, Anglia Ruskin University, Cambridge, UK; 4 Department of Ophthalmology, University Hospital of Bonn, Bonn, Germany; 5 Department of Ophthalmology, University Medical Center Hamburg-Eppendorf, Hamburg, Germany; 6 Institute of Ophthalmology, University College London, London, UK; 7 Moorfields Eye Hospital, London, UK

**Keywords:** choroideremia, autofluorescence, microperimetry, smooth zone, outcome measure

## Abstract

**Purpose:**

In patients with choroideremia, it is not known how smooth and mottled patterns on short-wavelength fundus autofluorescence (AF) imaging relate to retinal function.

**Methods:**

A retrospective case-note review was undertaken on 190 patients with choroideremia at two specialist centers for retinal genetics. Twenty patients with both smooth and mottled zones on short-wavelength AF imaging and concurrent mesopic microperimetry assessments were included. Mean retinal sensitivities within the smooth and mottled zones were compared between choroideremia patients, and identical points on mesopic microperimetry collected from 12 age-matched controls. Longitudinal analyses were undertaken at 2 and 5 years in a subset of patients.

**Results:**

In patients with choroideremia, mean retinal sensitivities at baseline were significantly greater in the smooth zone (26.1 ± 2.0 dB) versus the mottled zone (20.5 ± 4.2 dB) (*P* < 0.0001). Mean retinal sensitivities at baseline were similar in the smooth zone between choroideremia patients and controls (*P* = 0.054) but significantly impaired in the mottled zone in choroideremia compared to controls (*P* < 0.0001). The rate of decline in total sensitivity over 5 years was not significant in either the smooth or mottled zone in a small subset of choroideremia patients (*n* = 7; *P* = 0.344).

**Conclusions:**

In choroideremia, retinal sensitivity as determined by microperimetry correlates with patterns on AF imaging: retinal function in the smooth zone, where the retinal pigment epithelium is anatomically preserved, is similar to controls, but retinal sensitivity in the mottled zone is impaired.

**Translational Relevance:**

Patterns on AF imaging may represent a novel, objective outcome measure for clinical trials in choroideremia as a surrogate for retinal function.

## Introduction

Choroideremia (OMIM 303100) is an X-linked retinal degeneration that leads to severe sight loss usually by middle age. A loss-of-function mutation in the *CHM* gene causes deficiency in Rab escort protein 1 (REP1), essential for the regulation of intracellular vesicular trafficking.^1^ The retinal pigment epithelium (RPE) is likely to be the primary site of degeneration in choroideremia, which likely drives subsequent photoreceptor cell death, although a degree of independent photoreceptor and choriocapillaris loss has been observed.^2^^,^^3^ Clinically, the disease most commonly manifests in males with impaired night vision from childhood followed by progressive visual field restriction secondary to centripetal RPE and retinal degeneration. Visual acuity is typically affected late in the disease as the fovea becomes involved.^4^^,^^5^

Although there is currently no approved treatment for choroideremia, gene augmentation therapy has shown encouraging safety and efficacy in clinical trials.^6^^–^^10^ Relevant and objective outcome measures are necessary to identify patients suitable for inclusion, to select the optimal time for intervention, and to evaluate treatment effects. Although standard-luminance, high-contrast best-corrected visual acuity is generally considered by the regulatory authorities as a reliable marker of visual function in most retinal diseases, in patients with inherited retinal dystrophies, including choroideremia, this reliability can vary, reducing the precision to determine the true change in visual acuity. Natural history data in choroideremia suggest that visual acuity does not change significantly over a typical 12-month clinical trial period,^11^^,^^12^ limiting its value as a functional outcome measure, despite measurable improvements in early phase trials. Moreover, by the time the visual acuity significantly drops, the disease is at an advanced stage, and most of the retinal tissue may be beyond rescue and repair by gene therapy.^13^ Taken together, these observations encourage the validation of alternative outcome measures. If an objective structural outcome measure can be shown to be associated with preserved or improved retinal sensitivity, it may be of particular value in clinical trials as a surrogate endpoint.

**Figure 1.**
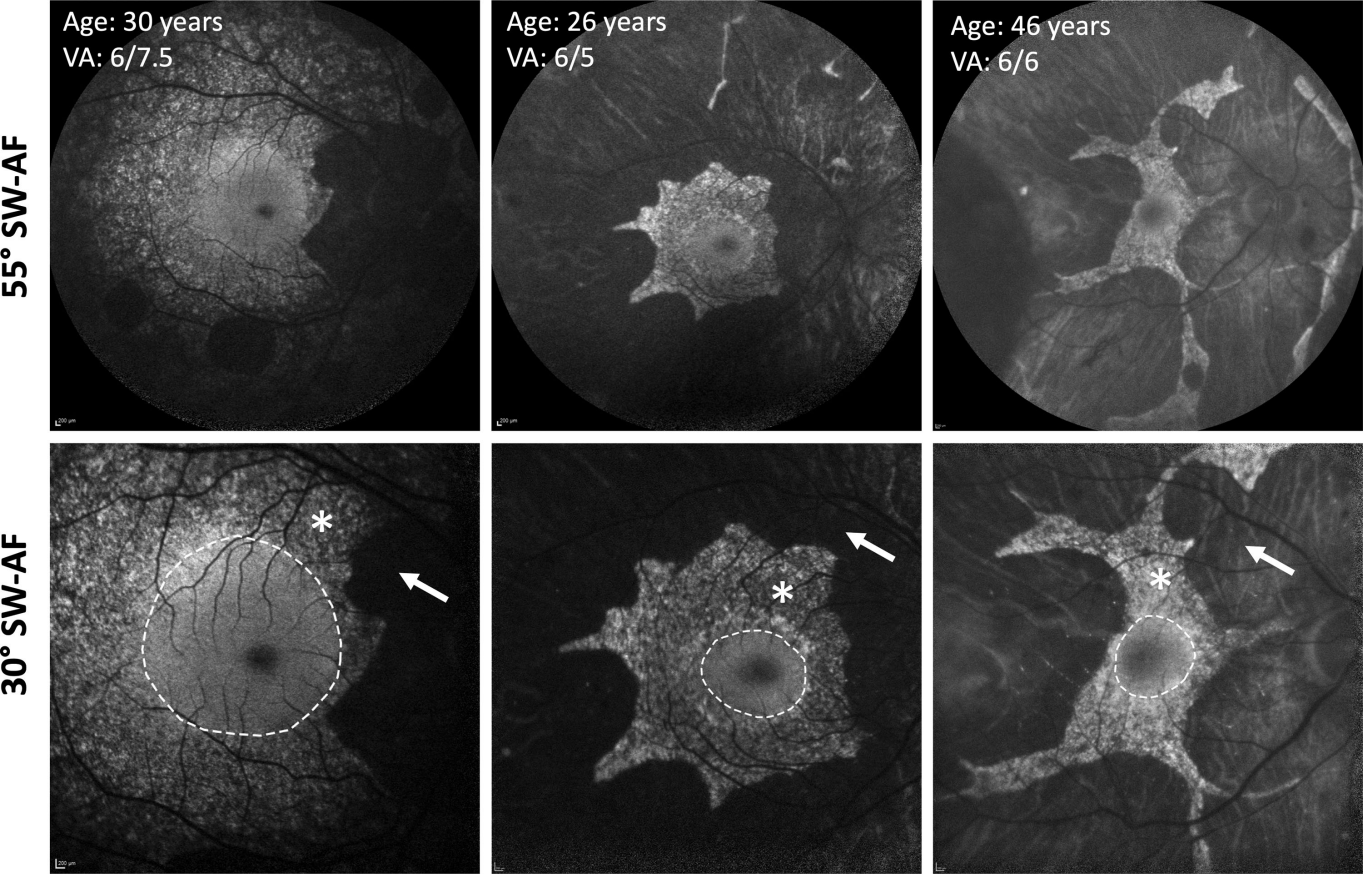
SW-AF images (top row 55°, bottom row 30°) of three patients with choroideremia demonstrate the three AF patterns: smooth (*dotted white line*) in the parafoveal region showing a homogeneous AF appearance; mottled (*asterisk*) showing a mixed granular hyper- and hypo-AF appearance; and atrophic (*arrow*) in the periphery showing confluent hypo-AF indicating loss of retinal pigment epithelium (RPE).

In a small proportion of patients with choroideremia, three distinct RPE patterns can be observed on short-wavelength autofluorescence (SW-AF) imaging, especially in the earlier disease stages: (1) smooth areas with a homogeneous AF pattern, (2) mottled areas with a coarse/granular mixed hyper- and hypo-autofluorescent textured appearance, and (3) atrophic areas that are hypo-autofluorescent (Fig. 1). These distinct appearances have been found to correlate with the integrity of the ellipsoid zone on optical coherence tomography (OCT) imaging, a biomarker of overlying photoreceptor health. Smooth AF zones were shown to contain a mostly intact ellipsoid zone and RPE, whereas mottled zones were associated with largely disrupted ellipsoid zones.^14^ Microperimetry (MP) enables assessment of central retinal sensitivity by combining scanning laser ophthalmoscopy with fundus-tracking software to allow accurate spatial mapping of retinal sensitivity at specific loci.^15^ Although a linear relationship has been identified between foveal sensitivity on MP and the distance from the center of the anatomical fovea to the nearest edge of degeneration on SW-AF imaging,^13^ it is not known how retinal function varies among the three imaging RPE patterns in choroideremia.

In this study, we investigated the relationship between patterns on SW-AF imaging (smooth and mottled) and retinal function as measured by MP in patients with choroideremia. We hypothesized that the smooth zone might be associated with preserved retinal function, which is impaired in the mottled zones.

## Materials and Methods

### Subjects

We conducted a retrospective review of SW-AF images of male patients with a clinical diagnosis of choroideremia confirmed on molecular genetic testing or a pedigree indicating X-linked inheritance when molecular testing could not be performed (*n* = 1). Patients were seen in specialist centers for retinal genetics: (1) Oxford Eye Hospital (Oxford, UK) as part the screening for a choroideremia gene therapy trial (NCT02407678) approved by the UK Research Ethics Committee (179453); and (2) Department of Ophthalmology, University of Bonn (Bonn, Germany) approved by the institutional review board at the University of Bonn. Age- and gender-matched healthy controls were recruited at the Oxford Eye Hospital as part of a visual function in inherited retinal disease study (ISRCTN24016133). Ethics approval for this study was granted by the UK Health Regulatory Authority (reference 20/WN/2083). All patients provided informed consent and the studies were conducted in accordance with the tenets of the Declaration of Helsinki.

**Figure 2.**
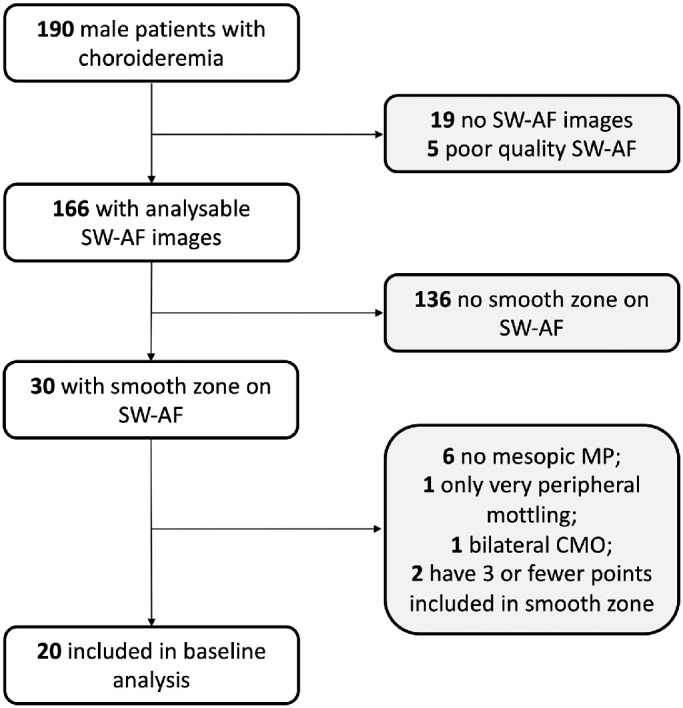
Flowchart showing the selection process for data included in the analysis. CMO, cystoid macular oedema; MP, microperimetry; SW-AF, short-wavelength autofluorescence.

For this analysis, we included only patients who had both smooth and mottled zones visible on SW-AF imaging and who had performed MP testing under mesopic conditions with a 10-2 grid as the test configuration. Patients who had ocular comorbidities that could have affected the measurement of retinal sensitivity were excluded. Tests that had three or fewer points on MP that fell within the smooth zone were excluded due to the significant potential for bias in the results (Fig. 2).

### Testing Protocols

SW-AF images had been obtained using standard operating protocols as part of routine clinical care (Heidelberg SPECTRALIS; Heidelberg Engineering, Heidelberg, Germany). When available, 30° images were used; otherwise, 55° images were used. Assessment of macular sensitivity was carried out using the MAIA Macular Integrity Assessment microperimeter (iCare; Icare Finland Oy, Vantaa, Finland) under mesopic test conditions using a 10-2 grid (68 points) centered on the fovea and following a 4-2 staircase thresholding strategy. No pupillary dilatation or formal dark adaptation was undertaken prior to testing.^16^^,^^17^ If patients performed more than one MP test, the second test was selected for analysis to account for the learning effect. All MP tests included in this study had stable fixation as defined by the MAIA output and had fixation losses of 25% or less. Retinal sensitivity values were reported in decibels (dB), with a 1-dB change corresponding to a 0.1 log unit change in stimulus luminance. Smooth and mottled zones on SW-AF images were delineated manually by the same grader to ensure consistency using Photoshop 22.4.2 (Adobe, Inc., San Jose, CA). The smooth zone was defined as an area of homogenous AF signal including the fovea. Mottled zones were identified by their more coarse/granular mixed hyper- and hypo-autofluorescent signal. Atrophic areas were defined as zones of absent AF (Fig. 1). Two delineation lines were drawn: one at the transition between the smooth and mottled zone and a second one at the transition between the mottled and atrophic zone. Using the same software, MP plots were then superimposed onto SW-AF images by resizing, stretching, and rotating the image as required to ensure accurate alignment of the imaging characteristics, particularly blood vessels and optic disc features, accounting for any differences in torsion and magnification between the two image testing modalities. Points within and on the delineation line were included in the relevant zone. Points falling on both lines for the smooth–mottled transition and mottled–atrophic transition were classified as mottled.

### Statistical Analysis

Statistical analysis was performed using Prism 9.3.1 (GraphPad Software, San Diego, CA) and R 4.2.1 (R Foundation for Statistical Computing, Vienna, Austria) using the lme4^18^ and flexplot^19^ functions. All statistical tests were two tailed, and *P* < 0.05 indicated significance. Normality was assessed using the Shapiro–Wilk test, and a bootstrapped distribution created from samples was used for non-normally distributed data.

**Figure 3.**
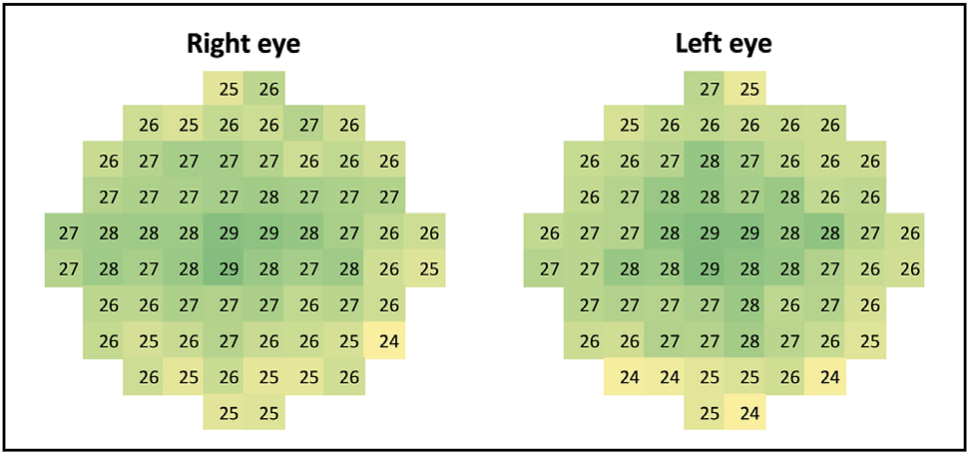
Mean retinal sensitivity on microperimetry in healthy controls. Microperimetry plots demonstrate point-by-point mean retinal sensitivity (dB) across the healthy control cohort (*n* = 12). Sensitivity is highest in the central macula, decreasing toward the peripheries of the tested region. The right eye was consistently tested first. Values are comparable between left and right eyes.

**Figure 4.**
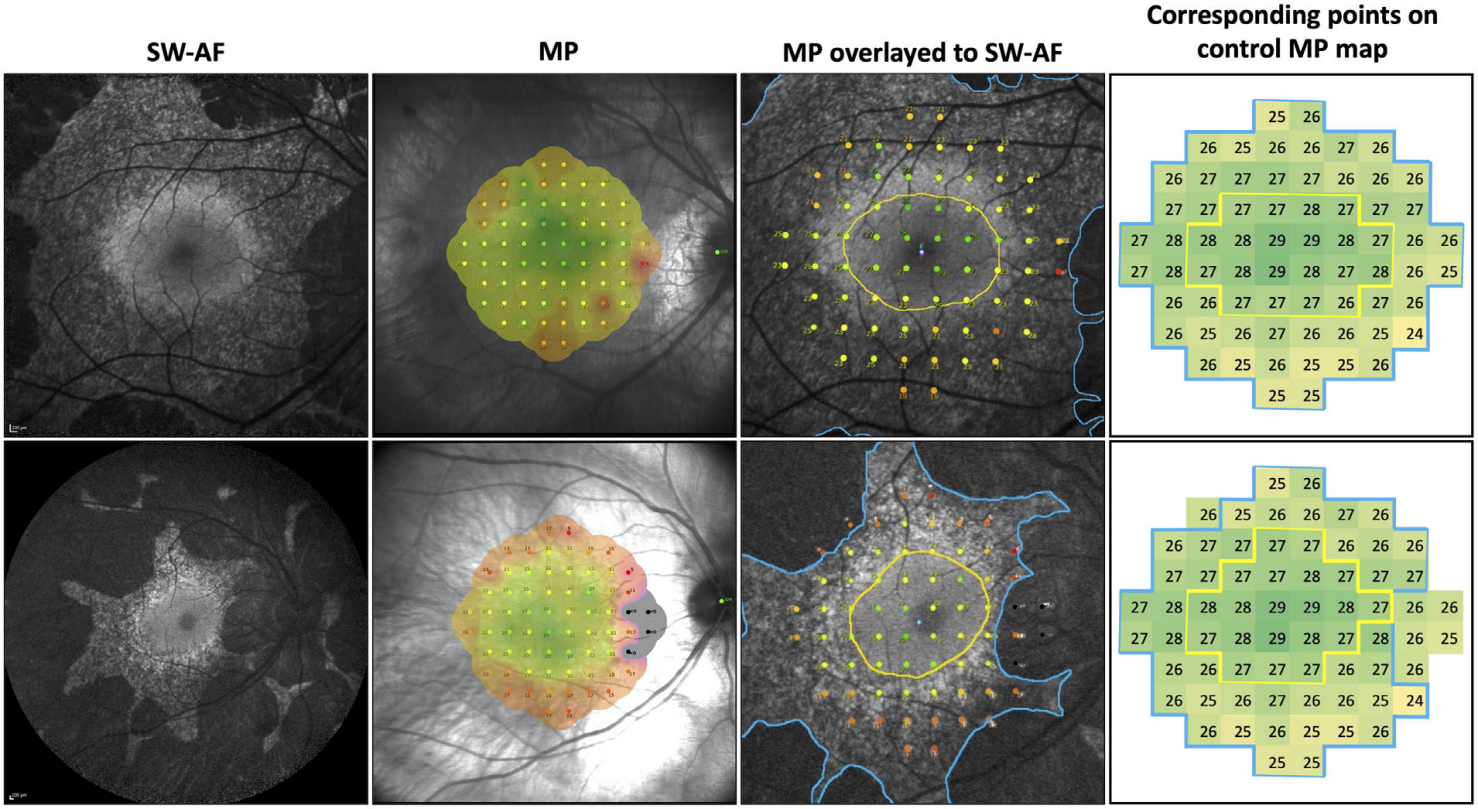
Method of structure–function analysis in choroideremia patients. Microperimetry (MP) maps are overlaid on short-wavelength autofluorescence (SW-AF) images by ensuring accurate alignment of retinal vessels. In the third column from the left, the *yellow line* delineates the smooth zone, and the *blue line* delineates the mottled zone. The fourth column demonstrates the points on the control MP maps corresponding to smooth (*yellow*) and mottled (*blue*) zones, used to calculate normalized data.

Mean sensitivities at baseline within the smooth and mottled zones on SW-AF for choroideremia patients and corresponding zones for controls were compared using a two-way analysis of variance (ANOVA), where the dependent variable was mean retinal sensitivity and the independent variables were a smooth versus mottled SW-AF pattern and choroideremia versus controls, with post hoc multiple-comparisons testing undertaken using the Šídák's test. For patients with choroideremia, mean MP sensitivity (dB) at baseline within the smooth and mottled zones was calculated as an average of the retinal sensitivity measurements for all points included within that area. Control data were used to calculate the mean sensitivity for each point on the MP plot (Fig. 3), enabling a calculation of normal mean sensitivity in healthy individuals for areas corresponding to the smooth and mottled zones in patients with choroideremia. Data were normalized by subtracting the mean retinal sensitivity for each zone in choroideremia patients from the mean retinal sensitivity for the corresponding zones in healthy controls (Fig. 4). This was necessary in order to adjust for the impact of eccentricity on retinal sensitivity, with the smooth zone being more central and the mottled zone more peripheral in the macula. Normalized data were compared using a paired *t*-test.

For the cross-sectional analysis, only data from right eyes were included.^20^ Further analyses were carried out on a subgroup of patients who had follow-up SW-AF and MP tests using the same protocols at 2 years and 5 years. For these analyses, data from the right eyes were included unless the patient had undergone gene therapy in their right eye, in which case the left eye was chosen.

Longitudinal analyses were carried out to evaluate the rate of change of the smooth and mottled zones over a 5-year follow-up period. Both zones were defined at baseline, and the same points were included at follow-up. Points that had a sensitivity of <1 dB at any time point were excluded from the analysis due to reaching the floor effect of the capability of the device.

A linear mixed model, with the dependent variable of total sensitivity and independent variables of time points (0, 2, and 5 years) and AF appearance (smooth vs. mottled), was fitted to evaluate preservation or decline of one zone relative to the other. Total summative sensitivity was used rather than mean sensitivity, as the latter relies on dividing by the number of points, a decreasing variable in a time-series analysis, creating discontinuities in the analysis due to changes in the number of seeing points present at each time point.

A point-by-point analysis was also performed where all the points within the retinal island were classified according to their appearance at baseline as smooth or mottled. The change over time for each individual point was calculated at the 2-year and 5-year intervals. These changes were compared between zones that were smooth versus mottled at baseline using the Mann–Whitney test, as these data were not normally distributed. This analysis enabled us to evaluate the extent of loss of retinal sensitivity during follow-up based on the baseline AF pattern (smooth or mottled).

## Results

### Subjects

Fundus autofluorescence images of a total of 190 patients with choroideremia were reviewed. Twenty patients met the inclusion criteria for the cross-sectional analysis as outlined in Figure 2. Further details including the results of molecular genetic testing and visual acuities of the patients included can be found in Supplementary Table S1. All patients were male. The mean age (± SD) for the group included in the analysis (31 ± 13 years) was significantly lower than the mean age of the whole sample screened (40 ± 15 years; *P* = 0.015) and that of the sample with no smooth zone on SW-AF (43 ± 15 years; *P* = 0.001). Seven patients had follow-up data available at 2 and 5 years. The 12 control subjects were males with a mean age similar to that of the choroideremia group (27 ± 8 years; *P* = 0.47).

### Cross-Sectional Analysis

**Figure 5.**
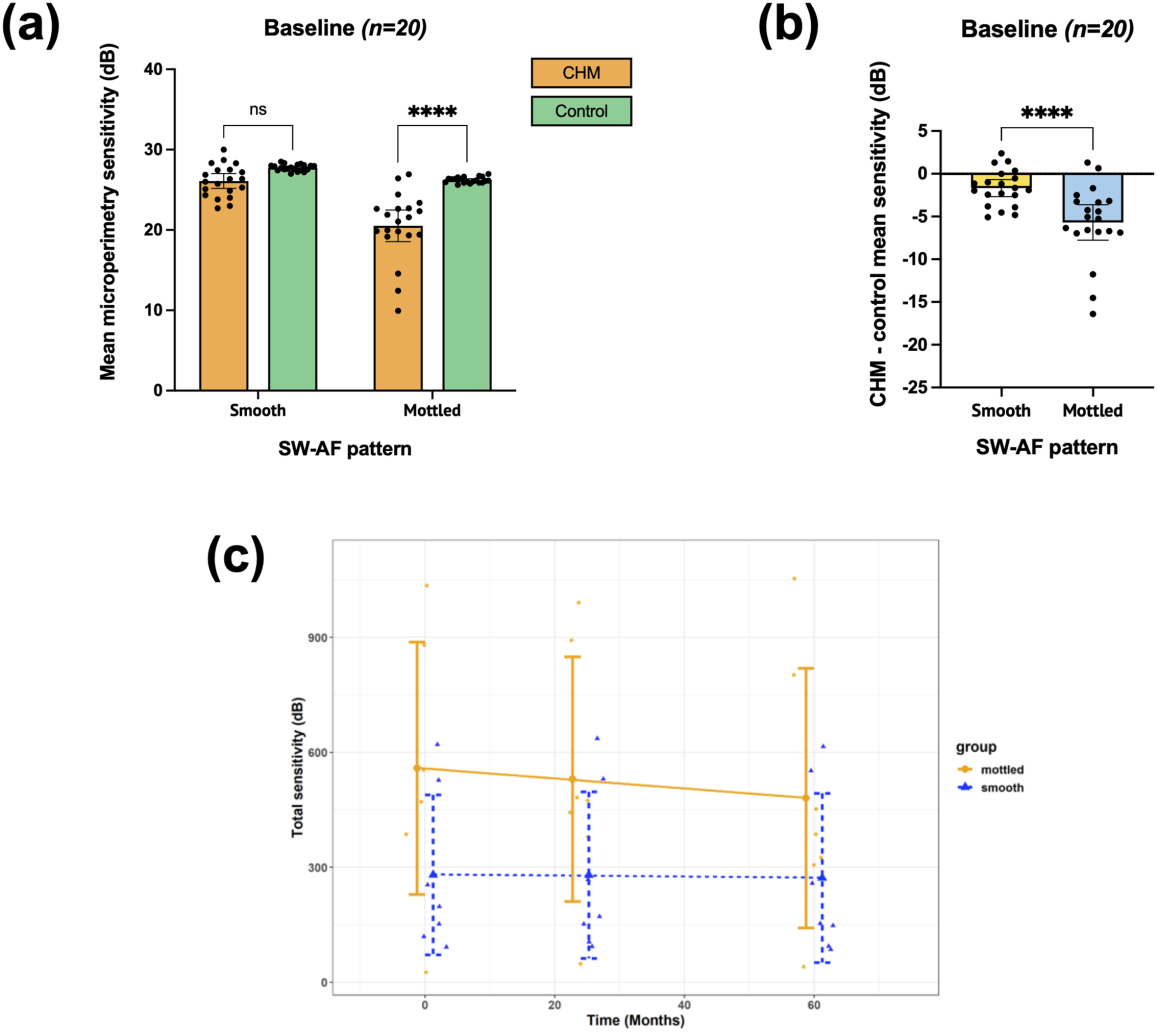
Analysis of retinal sensitivity in the smooth and mottled zones. (**a**) A comparison of mean microperimetry (MP) sensitivity within the smooth and mottled zones between patients with choroideremia (CHM) and controls at baseline. This shows a significantly greater sensitivity in the smooth compared to the mottled zone in patients with CHM. Mean retinal sensitivity in CHM is similar to the corresponding region in controls for the smooth zones but is significantly impaired for the mottled zones. (**b**) Normalized data for the smooth and mottled zones at baseline. This is calculated as SeCHM minus SeControl, where SeCHM is the mean sensitivity within each area in choroideremia and SeControl is the mean sensitivity of the corresponding points in controls. (**c**) Total sensitivity change over follow-up classified by short-wavelength autofluorescence (SW-AF) pattern (smooth and mottled). Linear mixed modeling shows that the rates of decline (slopes) in both the smooth and mottled zones are not significant, with no distinguishable difference between the two to any significant level. *Error bars*: 95% confidence intervals. ns, non significant; ****P < 0.0001.

On cross-sectional analysis (*n* = 20), in patients with choroideremia the average (± SD) MP mean sensitivities for the smooth and mottled zones were 26.1 ± 2.0 dB and 20.5 ± 4.2 dB, respectively. Both SW-AF pattern (smooth versus mottled), *F*(1, 76) = 45.90, *P* < 0.0001, and disease status (choroideremia versus control), *F*(1, 76) = 49.28, *P* < 0.0001, had a significant effect on MP sensitivity. Šídák's multiple comparisons testing showed that mean retinal sensitivity was similar to that of controls in the smooth zone (mean difference between choroideremia and controls was –1.67 dB; *P* = 0.054) but significantly impaired in the mottled zone (mean difference between choroideremia and controls was –5.70 dB; *P* < 0.0001) (Fig. 5A).

Normalized data were calculated in order to account for natural variation expected across the spatial locations of points tested within the MP grid (i.e., the smooth zone being more central and the mottled zone more peripheral). This confirmed a significantly greater sensitivity loss in the mottled zones compared to the smooth zones (*P* < 0.0001 on paired *t*-test) (Fig. 5B).

### Longitudinal Analysis

A total of 476 MP points were tested at each time point across the seven patients who had follow-up data (68 points per patient). At baseline, 78 MP points fell in areas with a smooth pattern on SW-AF, and 229 points fell in areas with a mottled pattern. One point was excluded from the smooth zone and 49 points from the mottled zones, as these had a sensitivity of <1 dB at any of the three time intervals, to avoid floor effects. Therefore, our analysis included 77 points in the smooth zone and 180 points in the mottled zone. Median sensitivities at baseline for the smooth and mottled zones were 25 dB (interquartile range [IQR], 23–27) and 23 dB (IQR, 21–23) respectively. A higher sensitivity decrease was found in points that at baseline had a mottled compared to a smooth SW-AF pattern. At 2 years, the median sensitivity loss in points that were smooth at baseline was 0 dB (–2 to 2 dB) compared to −2 dB (−4 to 1.75 dB) in those that were mottled at baseline (*P* = 0.005, Mann–Whitney test). Similarly, at 5 years, the median sensitivity loss in points that were smooth at baseline was 0 dB (−2 to 2 dB) compared to −2 dB (−6 to 0 dB) in points that were mottled at baseline (*P* < 0.0001, Mann–Whitney test). However, linear mixed modeling accounting for variations between patients and the repeated-measures nature of the data showed that the rates of decline over time of the total sensitivity in both the smooth and the mottled zones were not significant, with no difference between the two (*P* = 0.344) (Fig. 5C).

## Discussion

In this study, we show a correlation between retinal function as measured by MP and retinal structure as represented by patterns on SW-AF imaging in choroideremia. Within the residual island of retina, areas exhibiting smooth AF patterns demonstrate near-normal retinal sensitivity, whereas areas with a mottled, granular appearance are associated with measurably impaired retinal sensitivity compared to age-matched controls. Hence, in this disease, two AF patterns exist: an outer ring with a mottled pattern that indicates where RPE cells are surviving but are dysfunctional and an inner ring of smooth AF that indicates where RPE cells are still healthy and able to support overlying photoreceptors. The latter is evidenced by the maintained retinal sensitivity in the smooth zones.

These data support measurement of the smooth zone (preservation, or enlargement of) as a potential novel biomarker for clinical trials in choroideremia. Furthermore, validation of the functional significance of the smooth zone, as shown in this study, further strengthens its value as a surrogate for retinal function, as regulatory bodies generally prioritize functional outcome measures in pivotal clinical trials in the process of approval.^21^ This may be of value for patients with early choroideremia in whom normal visual acuity is expected and in whom a ceiling effect prevents further functional improvement using visual acuity as an outcome measure. Indeed, SW-AF imaging may be preferable to microperimetry in that it is an objective test and may be less variable than microperimetry, enabling a greater statistical power for the same number of study participants. The presence of the smooth zone may also be a valuable marker for clinical trial enrollment, where it could be used to highlight and include those participants who may have a greater therapeutic potential.

Choroideremia occurs due to a primary deficiency of REP1, resulting in defective prenylation of Rab proteins and impaired intracellular vesicular trafficking.^22^ Conditional knockout models have shown that the RPE is the primary affected cell type in choroideremia, although some independent photoreceptor and choriocapillaris degeneration is evident.^3^^,^^23^ This hypothesis is supported by multimodal retinal imaging studies, which appear to show surviving photoreceptors and choriocapillaris overhanging the edge of the island of remaining RPE. SW-AF imaging enables in vivo topographic mapping of the lipofuscin distribution in the RPE.^24^ The hypothesis that smooth zones may be areas of relatively preserved retina is supported by the previous description that these maintain a higher degree of outer retinal structural integrity, with a mostly intact ellipsoid zone and RPE, compared to mottled zones, which are associated with both ellipsoid zone and RPE disruption.^14^ It is also supported by recent findings with near-infrared (NIR)-AF in a choroideremia cohort, where the pattern of homogenous preservation of NIR-AF visually corresponded to areas of smooth appearance on SW-AF imaging in early disease stages.^25^ Structural disruption to the RPE in the mottled zone is also supported by a recent study that found subclinical polymegathism of the RPE cells in choroideremia using adaptive optics. This corresponds to the heterogeneous pattern of AF characteristic of the mottled zone.^26^

Our longitudinal analysis found that the rates of decline over time in both the smooth and mottled zone were not different in the small subset of patients for whom data were available (*n* = 7). The limit of the retinal island (i.e., mottled zone) has been shown to undergo exponential decay in a previous study.^27^ A key further study is required to model the change in area of the smooth zone over time and its rate of progression in choroideremia patients. This will subsequently enable identification of a time point to expect meaningful clinical change aiding adoption as an outcome measure in clinical trials.

Our study found that patients who had smooth zones were generally younger than those whose islands only exhibited mottled autofluorescence on SW-AF imaging. However, some individuals within our cohort did exhibit smooth zones into their fourth and fifth decades of life. Smooth zones always included the fovea, but the distance from the fovea to the border of smooth to mottled region was variable, as degeneration in choroideremia is not symmetrical around the fovea. Smooth zones were more often seen in patients with larger residual islands. Nonetheless, some patients had large islands with an entirely mottled appearance, whereas others had small islands with a well-demarcated smooth zone. Therefore, age and RPE island size alone do not appear to predict the presence of a smooth zone consistently, and a secondary mechanism responsible for AF pattern may be present.

The mechanism for the relative preservation of the smooth zone at the posterior pole is not yet known. As suggested by Stevanovic et al.,^14^ it may be that the increased metabolic support provided by the markedly thicker choroid and increased RPE density at the posterior pole may provide protection against the stress caused by REP1 deficiency. Additionally, the smooth zones correspond to areas of high cone photoreceptor density,^28^ where the cone mosaic is intact.^2^ Cone photoreceptors are believed to be less affected by REP1 deficiency, and the cone visual cycle is less dependent on the RPE than the rod visual cycle.^29^ These data support preservation of cone function until the underlying RPE is severely degenerated,^30^ in keeping with the preservation of visual acuity until the late disease stages^4^^,^^5^ and the colocalization of sensitivity loss and disorganization of the cone mosaic described by Tuten et al.^31^ using adaptive optics. Retinal changes are detectable from the early stages of disease when the RPE has not yet degenerated. Retinal thickening occurs, possibly secondary to Müller cell activation and hypertrophy in response to photoreceptor stress.^32^ This might explain the greater variability in retinal sensitivity and the slight overall lower mean sensitivity we observed in the smooth zone compared to equivalent areas in healthy controls. This may also be due to the inability to identify an intermediate stage between smooth and mottled on AF imaging due to its resolution. Further work is required to characterize rod function by dark-adapted scotopic MP to better understand the relative contributions of rods and cones to the visual function in smooth and mottled zones.

Some limitations of this study include the relatively limited sample size, especially for the longitudinal analysis. A further study to evaluate the longitudinal changes in the area of the smooth zone is required to better characterize its natural history. Second, the manual delineation of the smooth zone could be considered subjective. However, the points on the 10-2 MP grid are relatively spaced apart (2° spacing); slight variation in the contours of the smooth zone would be critical for any area change analysis but is unlikely to lead to changes to the MP points included. Third, not all patients and controls in the study had two MP tests; therefore, the learning effect was not relevant for all subjects. Nonetheless, this could make the findings more directly applicable to routine clinical practice. Another factor to take into consideration is that the MP fundus tracking frequency (25 Hz) is likely not to fully compensate for all microsaccades that occur during the stimulus presentation.^33^ Consequently, there is a small amount of variability of the exact retinal locus being tested that might not exactly correspond to the point represented on the MP plot. The MP coefficient of repeatability for mean sensitivity in choroideremia has been described as less than 2 dB.^13^^,^^33^ Therefore, the difference between mean retinal sensitivity in the smooth versus mottled zones that we observed at baseline (just over 5 dB) is likely to be clinically meaningful. On the other hand, the reported point-wise sensitivity repeatability of around 5 dB^33^ makes the statistically significant difference between smooth and mottled zones in our longitudinal point-by-point analysis less likely to be clinically significant, in keeping with the lack of difference in the rates of decline found by the linear mixed model. The variability in results is especially marked at the transition zone between mottled and atrophic regions, where there is a sharp decline in sensitivity with a cliff-edge effect. This is supported by the higher test–retest variability at the border of the degeneration observed by Dimopoulos et al.^33^ and by the findings by Wyatt et al.^34^ that test–retest variability depends in a significant part on small fixational eye movements that shift the test stimulus on the retina. This introduces challenges when monitoring sensitivity changes at these transitional zones, which consequently affects calculations of mean sensitivity. For longitudinal analyses, the slow rate of degeneration might mean that it could take many years to observe clinically significant changes using the more variable point-wise analyses. Hence, global metrics and volumetric analyses may be more sensitive measures with better repeatability.^33^^,^^35^

In summary, we found a correlation between retinal patterns on SW-AF and retinal function on MP. Areas with a smooth autofluorescence exhibit retinal sensitivity that is not significantly different from controls, whereas areas with granular, mottled autofluorescence are associated with significantly impaired function. The functional preservation we have observed in the smooth zone is an important validation step toward a novel outcome measure in choroideremia and supports the findings of a previous study in which OCT imaging findings correlated with SW-AF imaging patterns.^14^^,^^25^ Patterns on SW-AF may therefore be used as a surrogate for retinal function in both routine clinical care and clinical trials for the evaluation of novel therapies, particularly because AF is more widely available. This has additional implications for gene therapy in choroideremia, as the smooth zone area on SW-AF might be used as a functionally validated structural biomarker that is relevant to patients with early disease, in whom measurement of visual acuity is limited by a ceiling effect. Moreover, because transduction and rescue require areas of surviving RPE and photoreceptor cells in order to restore prenylation activity,^36^ targeting the smooth zone with subretinal delivery may therefore be more effective. The structural degeneration and functional reduction already present in the mottled zone possibly indicate a state of stress in the RPE, which may be more difficult to rescue. Further work is required to characterize the natural history of changes in the smooth zone area and sensitivity over time.

## Supplementary Material

Supplement 1
