# Supplementary material for: Correlation Between Fundus Autofluorescence Pattern and Retinal Function on Microperimetry in Choroideremia
Source: Transl Vis Sci Technol. 2023 Sep 29;12(9):24. doi: 10.1167/tvst.12.9.24 (PMC10547012; doi:10.1167/tvst.12.9.24)
Supplement: Supplement 1 [file tvst-12-9-24_s001.pdf]

Study ID	Age (years)	Baseline VA RE (m)	Baseline VA LE (m)	Mutation	Protein change	Mean Sensitivity (dB) smooth zone	Mean Sensitivity (dB) mottled zone
1	29	6/7.5	6/7.5	c.808C>T	p.(Arg270Ter)	24.00	19.85
2	26	6/6	6/6	c.808C>T	p.(Arg270Ter)	23.80	19.97
3	45	6/6	6/6	c.49+2dupT	Splice donor site	23.00	12.44
4	47	6/6	6/6	c.808C>T	p.(Arg270Ter)	22.71	19.16
5	18	6/7.5	6/9	c.1079del	Frameshift	27.44	23.37
6	27	6/5	6/5	c.886del	p.(Met296Ter)	30.00	24.42
7	23	6/7.5	6/9	c.819+1G>A	Splice donor site	28.33	22.33
8	20	6/6	6/4	c.179del	Frameshift	26.35	22.63
9	36	6/5	6/4	c.940+3del	Splice donor site	25.75	22.70
10	15	6/4	6/6	c.1780del	Frameshift	27.18	19.82
11	17	6/3	6/3	c.703-1 727delinsTTAGA	Intron 5	25.29	21.90
12	27	6/4	6/4	c.737_741	Frameshift	24.33	19.41
13	55	6/7.5	6/7.5	c.1770+1G>C	Splice donor site mutation	28.70	26.42
14	20	6/4	6/5	c.940+3del	Splice donor site	28.30	26.93
15	22	6/4	6/5	c.315-1del	Splice acceptor site	26.00	21.04
16	26	6/5	6/7.5	c.715C>T	p.(Arg239Ter)	25.10	19.33
17	46	6/6	6/6	c.1762_1765del	Frameshift	26.33	9.93
18	57	6/7.5	6/7.5	c.653C>G	p.(Ser218Ter)	24.88	22.63
19	27	6/7.5	6/6	c.757C>T	p.(Arg253Ter)	26.88	14.57
20	40	6/6	6/6	Refused testing. Clinical diagnosis with X-linked pedigree.		27.50	21.57
